# P16 Analysis of antibiotic resistance in wounded servicemen with infectious complications

**DOI:** 10.1093/jacamr/dlae136.020

**Published:** 2024-08-23

**Authors:** Oksana Viltsaniuk, Viktor Syvak, Nataliia Tkachuk

**Affiliations:** National Pirogov Memorial Medical University, Vinnytsia, Ukraine; Military Medical Clinical Centre of the Central Region, Ukraine; Military Medical Clinical Centre of the Central Region, Ukraine

## Abstract

**Background:**

The problem of antibiotic resistance has now become a global challenge. Infectious complications are the cause of death in 80% of wounded soldiers whose deaths occur late after a military injury. This confirms that the fight against wound infection is one of the priority tasks in the treatment of combat trauma.

**Objectives:**

To investigate the species composition and susceptibility of microorganisms in servicemen with gunshot wounds and severe infectious complications.

**Materials and methods:**

The study analysed 34 case histories of wounded servicemen with infectious complications (sepsis, pneumonia).

**Results:**

Sixty-eight samples of materials were taken, of which 56 samples of biomaterial were isolated from wounds, blood and sputum, and pathogens were isolated. A monoculture was isolated in 85% of cases, in 10% of cases there was a combination of *Staphylococcus aureus* and *Klebsiella pneumoniae*, and in 5% of cases different triple combinations of *S. aureus* with *Staphylococcus lentus*, *Enterobacter*, *Proteus* or *Lelliottia amnigena* were identified. Most commonly detected were *K. pneumoniae* (43%) and *S. aureus* (21%), followed by equal proportions of *Enterobacter* and *Proteus* (both 9%), *Bacillus* spp. (7%) and, in rare cases, *L. amnigena*, *S. epidermidis*, *S. haemolyticus* and *S. lentus* were isolated (1% each, respectively). Considering that *S. aureus* and *K. pneumoniae* were the most frequently isolated pathogens, we analysed their susceptibility. *K. pneumoniae* was resistant to cefazolin in all cases, and the highest resistance was found to amikacin, amoxicillin, ciprofloxacin, cefepime and piperacillin/tazobactam. The highest levels of susceptibility were found for levofloxacin and meropenem. Susceptibility analysis of *S. aureus* revealed that this pathogen was not susceptible to azithromycin, amoxicillin, levofloxacin, moxifloxacin, ofloxacin, piperacillin/tazobactam or cefazolin. Linezolid had the highest susceptibility of 90%.

**Conclusions:**

The choice of antibiotic therapy for wounded soldiers should take into account the possibility of infection with highly virulent pathogens that can cause secondary purulent complications. In addition, it should be noted that due to the phasing of care from the battlefield to tertiary care, the risk of developing resistance increases as pathogens constantly migrate and change their susceptibility to antibiotics.

